# Prognostic value of nutritional and inflammatory indicators in females with esophageal squamous cell cancer: A propensity score matching study

**DOI:** 10.3389/fgene.2022.1026685

**Published:** 2022-10-31

**Authors:** Yuping Li, Huaichao Luo, Bo Ye, Kaijiong Zhang, Chang Liu, Ruiling Zu, Sujiao Ni, Qiao He, Lubei Rao, Qifeng Wang, Dongsheng Wang

**Affiliations:** ^1^ Department of Clinical Laboratory, Sichuan Cancer Hospital and Institution, Sichuan Cancer Center, School of Medicine, University of Electronic Science and Technology of China, Chengdu, China; ^2^ Department of Radiation Oncology, Sichuan Cancer Hospital and Institution, Sichuan Cancer Center, School of Medicine, University of Electronic Science and Technology of China, Chengdu, China

**Keywords:** propensity score matching, serum total cholesterol, esophageal squamous cell cancer, female, nutritional and inflammatory indicators

## Abstract

**Background:** Esophageal squamous cell cancer (ESCC) is a disease with a male predominance. Accordingly, the applicability of prognostic indicators values previously set for the general population with ESCC has not been reported for determining the physical state in females.

**Methods:** Patients with ESCC were pooled from 2009 to 2017 at Sichuan Cancer Hospital. We determined the differences in the nutritional and inflammatory indicators between gender by sex-stratified survival analysis in all cohorts (*n* = 2,660) and matching cohorts (*n* = 483 pairs) separately. Propensity score matching (PSM) was employed to eliminate selection bias between genders. We further performed the prognostic value of total cholesterol (TC) by subgroup analysis in the female cohort. The area ROC curve was used to assess the predictive performance of TC in females.

**Results:** There were a total of 2,660 patients with ESCC, of whom 2,173 (81.7%) were male and 487 (18.3%) were female. Before PSM, the prognostic nutritional index was an independent factor for OS in males but not in females. For cohort with or without matching, TC was an independent prognostic factor in females not for males. Furthermore, female patients with high TC level had significant poor OS in stages III and IV. The AUCs of TC were 0.63 and 0.70 for predicting 3- and 5-year OS, respectively.

**Conclusion:** Based on a much larger cohort, we confirmed that gender was a significant prognostic factor for ESCC patients. Interestingly, we found a significant difference in TC related to ESCC prognosis between genders. Collectively, TC might be an independent prognostic factor in females with ESCC.

## Introduction

According to the report of Global Cancer Statistics 2020, esophageal cancer ranks seventh in terms of incidence and sixth in mortality overall (Sung et al., 2021). In China, esophageal cancer is the fifth in terms of incidence and fourth in mortality overall among all malignant tumors (Hou et al., 2019). In addition, as the predominant histopathological type, esophageal squamous cell cancer (ESCC) covers more than 90% of all esophageal cancer cases in China (Lin et al., 2013; Arnold et al., 2015). Male predominance in ESCC had been well established across international cohorts (Sung et al., 2021). Though many studies with different sample sizes and research designs have been performed on the association between various clinic biomarkers and the prognosis of ESCC, the prognostic values of these prognostic biomarkers in the female with ESCC remain unclear.

At present, numerous nutritional status predictors and inflammation biomarkers, such as platelet to lymphocyte ratio (PLR), systemic immune-inflammation index (SII), and prognostic nutritional index (PNI) had been associated with prognosis in esophageal cancer (Sharaiha et al., 2011; Feng et al., 2017; Okadome et al., 2020). Sex difference in terms of potential risk for esophageal cancer was considered significant (Huang and Yu, 2018). Therefore, with men accounting for approximately 80% of all cases in prior studies, the results might represent more males rather than females. At present, no studies regarding these prognostic predictors in female patients with ESCC have been reported. Therefore, we aimed to compare the differences between clinical prognostic predictors and outcomes in sex and determine the prognostic values of these variables in the female cohort in our study.

Serum total cholesterol (TC) and triglycerides as prognostic indicators had been reported in various cancers (Asano et al., 2008; Mondul et al., 2011; Wulaningsih et al., 2012; Wulaningsih et al., 2015; Radišauskas et al., 2016). But studies about the relationship between lipids and ESCC were limited and controversial. Chen et al. (2017) indicated that low serum TC level was a predictive factor for poor survival in esophageal cancer patients who underwent esophagectomy. However, due to the small sample size in prior studies, the effects of TC and triglyceride levels on the incidence of ESCC have not been well elucidated. Moreover, less research has been published previously regarding TC in female patients with ESCC.

Esophageal squamous cell cancer is a disease with a male predominance. In this study, we aimed to investigate and verify the prognostic value of the PNI, PLR, SII, and TC in a female cohort of patients with surgically resected ESCC. Also, the results might provide some new clues for further investigations in therapy and prognosis of ESCC by sex-stratified in the future.

## Subject and methods

### Study procedure

**FIGURE 1:**
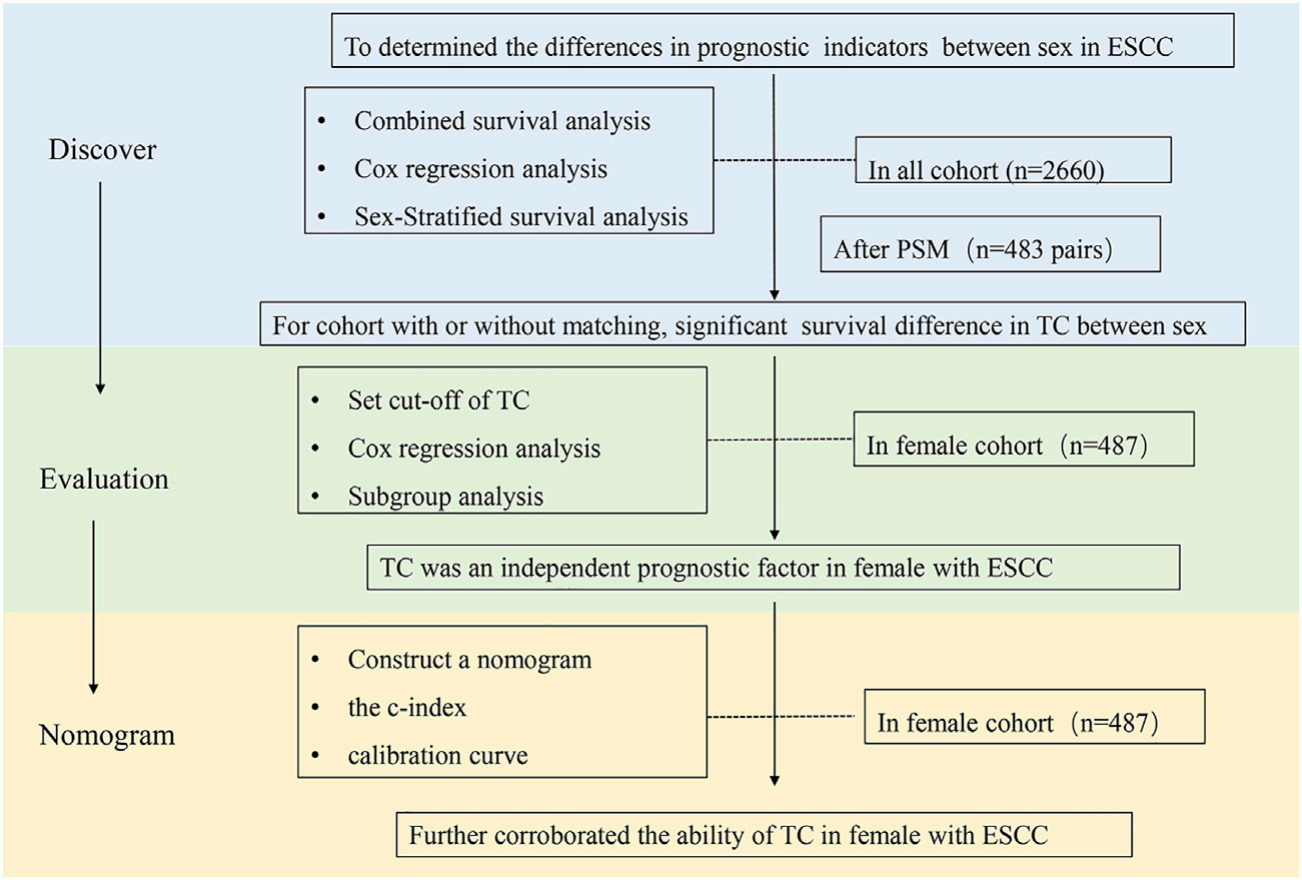
The flow chart of this study.

First, we determined the differences in the clinical prognostic indicators between gender by sex-stratified survival analysis in all cohorts (*n* = 2,660) and matching cohorts (*n* = 483 pairs) separately. We found there was a significant survival difference in TC between sex for the cohort with or without matching. Second, 487 female patients were separated into two groups according to the optimal cut-offs of TC. Subsequently, Cox regression analysis and subgroup analysis were employed to further evaluate the prognostic value of TC in females. The result showed TC was an independent prognostic factor in females with ESCC. Subsequently, we established a nomogram based on TC combined with TNM stage, neural invasion, and tumor diameter. The c-index and calibration curve were used to further corroborate the ability of the TC indicator (Figure 1).

### Subject

This was a retrospective clinical study and approved by Sichuan Cancer Hospital (SCCHEC-02-2020-015). A total of 2,660 patients with ESCC were pooled from 2009 to 2017 at Sichuan Cancer Hospital in this study. All patients with ESCC were histologically confirmed by surgery and pathology. The exclusion criteria were: surgery in the other hospital; lack of other clinicopathological or laboratory parameters; inadequate follow-up information. The pathological stage was classified according to the eighth edition of the American Joint Committee on Cancer TNM classification system (Rice et al., 2017). The preoperative blood data of patients were analyzed in the clinical laboratory at Sichuan Cancer Hospital within 1 week prior to surgery. Patients were followed up regularly as outpatients every three to 6 months for the first 2 years after surgery and then annually thereafter until death or the end of the study period.

### Data

We retrospectively collected the patient's clinicopathological characteristics from medical records. These characteristics included age, TNM stage, tumor grade, surgical margin, tumor location, postoperative adjuvant treatment, neural invasion, vascular invasion, tumor diameter, and overall survival (OS). Data on the preoperative laboratory examination were extracted from the clinical laboratory. Complete blood count was measured with Mindray BC-6800 (Shenzhen, China) using the manufacturer’s kit. Blood biochemical examination was performed using the Beckman Coulter AU5800 analyzer (Brea, CA) and manufacturer’s kits.

### Statistical analysis

Categorical variables were presented as numbers and percentages, and groups were compared using the χ2 test or Fisher exact test. According to the normal distribution, continuous variables were described as means and standard deviations (SDs) and compared by sex using a *t*-test when appropriate. Variables that did not follow a normal distribution were expressed as the median and interquartile range (IQR) and differences were identified by Mann–Whitney U test. Categorical variables were performed as numbers and proportions and groups were compared using Chi-square or Fisher exact test. The survival time distribution was performed by the Kaplan–Meier method, and the comparisons were carried out using the log-rank test. We used the multivariate Cox proportional hazard model to adjust for the potential confounds regarding clinical and pathological variables. All the associations were estimated first in general and then estimated among males and females separately. Propensity score matching (PSM) was employed to reduce the bias from baseline confounding variables. The optional cut-off value of TC was determined by constructing receiver operating characteristic (ROC) curves. The area ROC curve (AUC) was used to assess the predictive performance of TC. Ultimately, those analyses were performed using SPSS 25.0 (IBM, Armonk, NY).

A novel nomogram was formulated based on the significant factors of multivariate Cox regression analysis. The calibration curve was used to calibrate the nomogram. Also, the concordance index (c-index) was used to quantify the discrimination performance of the nomogram. The nomogram, the c-index, and the calibration curve were implemented by R software (version 4.2.1). *p* value less than 0.05 was considered statistically significant.

## Results

### Baseline characteristics and combined survival analysis

There were a total of 2,660 patients with ESCC, of whom 2,173 (82%) were male and 487 (18%) were female. The baseline characteristics of the examined cases were summarized in Table 1. Sex was significantly associated with age (*p* = 0.03), TNM stage (*p* < 0.01), tumor grade (*p* = 0.04), tumor location (*p* < 0.01), postoperative adjuvant treatment (*p* < 0.01), neural invasion (*p* < 0.01), vascular invasion (*p* < 0.01), tumor diameter (*p* < 0.01). There were no significant differences in the surgical margin (*p* > 0.05) between males and females. The univariate and multivariate Cox regression analysis showed that sex, TNM stage, surgical margin, vascular invasion, neural invasion, tumor grade, tumor diameter, and PNI were independent prognostic factors in all patients (*p* < 0.05) (Table 2).

**TABLE 1:** Patient characteristics of total cohort.

Characteristics	Overall	Male	Female	*P*
	(*n* = 2660)	(*n* = 2173)	(*n* = 487)	
Age Median (IQR)	62.00 (57.00–67.00)	62.00 (57.00–67.00)	63.00 (58.00-68.00)	**0.02**
TNM stage				<**0.01**
0	58 (2.2)	38 (1.7)	20 (4.1)	
I	249 (9.4)	182 (8.4)	67 (13.8)	
II	852 (32.1)	687 (31.6)	165 (33.9)	
III	1178 (44.3)	979 (45.1)	199 (40.9)	
IV	323 (12.1)	287 (13.2)	36 (7.4)	
Tumor Grade				**0.04**
Poorly	1113 (41.8)	885 (40.7)	228 (46.8)	
Moderate	1060 (39.9)	890 (41.0)	170 (34.9)	
Well	487 (18.3)	398 (18.3)	89 (18.3)	
Tumor location				<**0.01**
Lower_	572 (21.5)	520 (23.9)	52 (10.7)	
Middle	1418 (53.3)	1171 (53.9)	247 (50.7)	
Upper	670 (25.2)	482 (22.2)	188 (38.6)	
Postoperative adjuvant treatment				<**0.01**
No	1421 (53.4)	1135 (52.2)	286 (58.7)	
Yes	1239 (46.6)	1038 (47.8)	201 (41.3)	
Surgical margin				0.13
R0	2525 (94.9)	2054 (94.5)	471 (96.7)	
R1	90 (3.4)	80 (3.7)	10 (2.1)	
R2	45 (1.7)	39 (1.8)	6 (1.2)	
Neural invasion				<**0.01**
No	2157 (81.1)	1725 (79.4)	432 (88.7)	
Yes	503 (18.9)	448 (20.6)	55 (11.3)	
Vascular invasion				<**0.01**
No	2196 (82.6)	1773 (81.6)	423 (86.9)	
Yes	464 (17.4)	400 (18.4)	64 (13.1)	
Tumor Median (IQR), cm	4.00 (2.80–5.00)	4.00 (3.00–5.00)	3.50 (2.50–4.80)	<**0.01**
TC Median (IQR), mmol/L	4.83 (4.25–5.47)	4.22 (4.77–5.42)	5.05 (4.45–5.69)	<**0.01**
TG Median (IQR), mmol/L	1.12 (0.87–1.43)	1.10 (0.86–1.41)	1.18 (0.94–1.53)	<**0.01**
PNI Median (IQR)	50.55 (47.40–53.65)	50.37 (47.15–53.53)	51.38 (48.60–54.25)	<**0.01**
PLR Median (IQR)	117.67 (88.52–153.92)	118.99 (90.03–155.23)	110.19 (83.90–148.31)	<**0.01**
SII Median (IQR)	450.94 (300.85–671.23)	471.46 (313.37–693.65)	381.86 (249.98–584.65)	<**0.01**

The bold *P* value less than 0.05 was considered statistically significant; TNM, tumor node; IQR, interquartile range; TC, total cholesterol; SII, systemic immune-inflammation index; PNI, prognostic nutritional index; PLR, platelet-lymphocyte ratio; TG, triglycerides.

**TABLE 2:** Univariate and multivariate analysis of overall survival in the overall cohort.

Variables	Overall cohort (*n* = 2,660)			
	Univariate analysis		Multivariate analysis	
	HR (95% CI)	P	HR (95% CI)	P
Age (≦60 vs. > 60)	0.94 (0.84–1.06)	0.30		
Gender (male vs. female)	1.70 (1.45–2.0)	<0.01	1.45 (1.23–1.70)	**< 0.01**
TNM stage (0/1/2 vs. 3/4)	0.31 (0.27–0.35)	<0.01	0.37 (0.33–0.43)	**< 0.01**
Surgical margin (R0 vs. R1/R2)	0.56 (0.45–0.69)	<0.01	0.72 (0.58–0.89)	**< 0.01**
Location (upper vs. middle, lower)	1.06 (0.94–1.20)	0.34		
Vascular invasion (no vs. yes)	0.54 (0.47–0.62)	<0.01	0.73 (0.64–0.84)	**< 0.01**
Neural invasion (no vs. yes)	0.63 (0.55–0.72)	<0.01	0.83 (0.72–0.95)	**< 0.01**
Tumor grade (moderate, poorly vs. well)	1.44 (1.23–1.68)	<0.01	1.26 (1.08–1.48)	**< 0.01**
Postoperative adjuvant treatment (no vs. yes)	1.03 (0.92–1.15)	0.67		
Tumor diameter (continuous)	1.1241.10–1.15)	<0.01	1.07 (1.05–1.11)	**< 0.01**
TC (continuous)	0.94 (0.88–0.99)	0.03	0.99 (0.93–1.05)	0.73
TG (continuous)	0.93 (0.87–1.00)	0.06		
PNI (continuous)	0.97 (0.96–0.99)	<0.01	0.98 (0.97–1.00)	**0.02**
PLR (continuous)	1.00 (1.00–1.00)	<0.01	1.00 (1.00–1.00)	0.64
SII (continuous)	1.00 (1.00–1.00))	<0.01	1.00 (1.00–1.00)	0.75

The bold *P* value less than 0.05 was considered statistically significant. HR, hazard ratio; CI, confidence interval; TNM, tumor node; SII, systemic immune-inflammation index; PNI, prognostic nutritional index; PLR, platelet-lymphocyte ratio; TC, total cholesterol; TG, triglycerides.

### Baseline characteristics and combined survival analysis after propensity score matching

**FIGURE 2:**
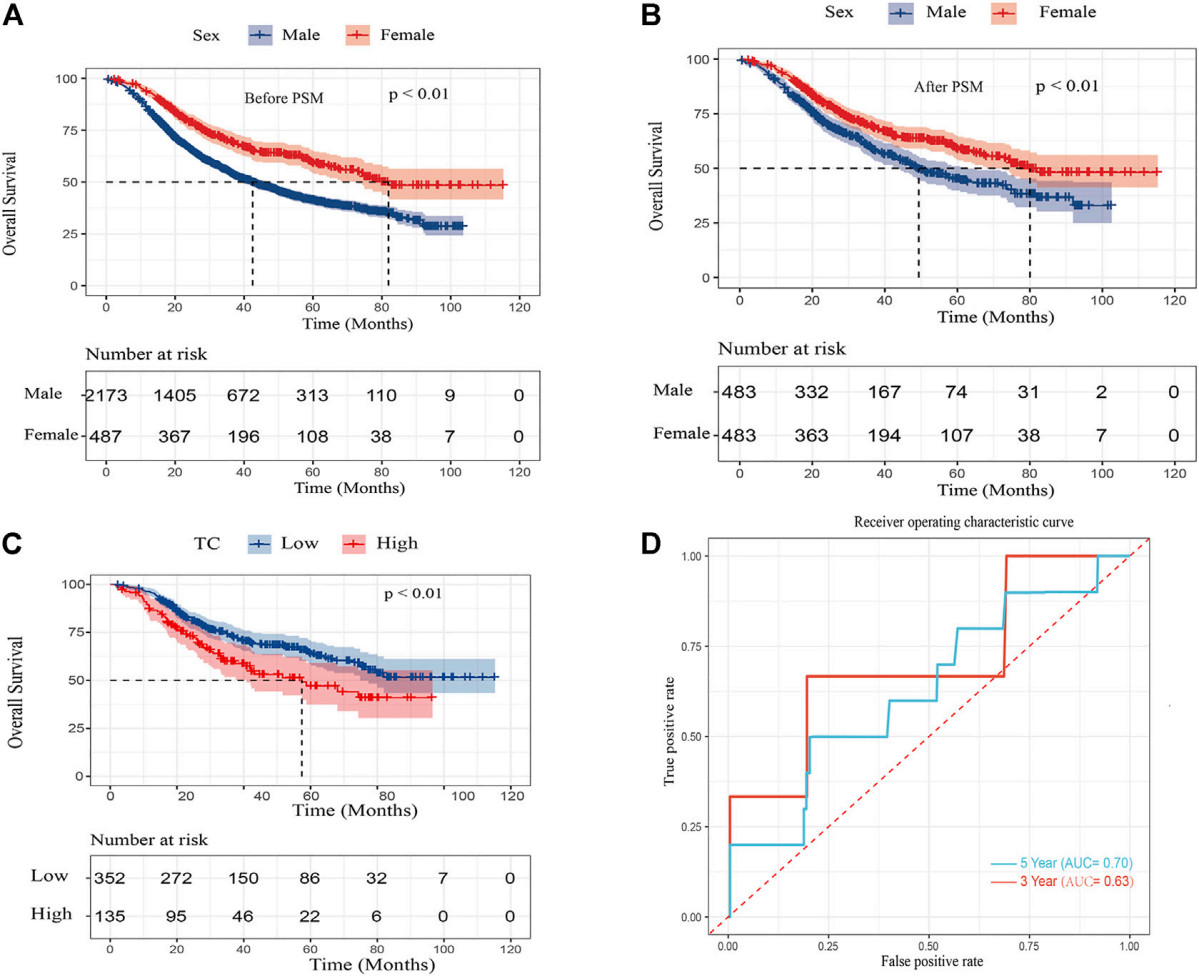
Kaplan–Meier curves for overall survival in ESCC patients **(A)** after PSM by sex **(B)** and overall survival in female ESCC patients by TC **(C)** and area under the curves (AUCs) of TC for 3- and 5-year survival rates in the female cohort **(D)**.

To balance confounding variables and eliminate selection bias, The PSM was performed by a 1:1 matching protocol with caliper <0.01 and no replacement in the male and female groups. After PSM, a total of 483 patient pairs were extracted and there were no significant differences in baseline characteristics between sex (*p* > 0.05 for all) (Table 3). After PSM, the multivariate Cox regression analysis indicated that age, gender, TNM stage, vascular invasion, and tumor diameter were independent prognostic factors (*p* < 0.05 for all) (Table 4). For cohorts with or without matching, the Kaplan–Meier analysis showed females had a significantly longer OS (log-rank *p* < 0.01) than males (Figures 2A,B).

**TABLE 3:** Patient characteristics of the total cohort after PMS.

Characteristics	Overall (*n* = 966)	Male (*n* = 483)	Female (*n* = 483)	P
Age median (IQR)	63.00 (58.00–68.00)	63.00 (58.00–68.00)	63.00 (58.00–69.00)	0.49
TNM stage				**0.95**
0	36 (3.7)	19 (3.9)	17 (3.5)	
I	131 (13.6)	65 (13.5)	66 (13.7)	
II	319 (33.0)	154 (31.9)	165 (34.2)	
III	407 (42.1)	208 (43.1)	199 (41.2)	
IV	73 (7.6)	37 (7.7)	36 (7.5)	
Tumor grade				0.60
Poorly	464 (48)	239 (49.5)	225 (48.5)	
Moderate	324 (33.5)	155 (32.1)	169 (35.0)	
Well	178 (18.4)	89 (18.4)	89 (18.4)	
Tumor location				**0.94**
Lower	104 (10.8)	52 (10.8)	52 (10.8)	
Middle	487 (50.4)	241 (49.9)	246 (50.9)	
Upper	375 (38.8)	190 (39.3)	185 (38.3)	
Postoperative adjuvant treatment				**0.29**
No	589 (61)	303 (62.7)	286 (59.2)	
Yes	377 (39)	180 (37.3)	197 (40.8)	
Surgical margin				0.05
R0	917 (94.9)	450 (93.2)	467 (96.7)	
R1	31 (3.2)	21 (4.3)	10 (2.1)	
R2	18 (1.9)	12 (2.2)	6 (1.2)	
Neural invasion				**0.62**
No	850 (88.0)	422 (87.4)	428 (88.6)	
Yes	116 (12.0)	61 (12.6)	55 (11.4)	
Vascular invasion				**0.78**
No	838 (86.7)	417 (86.3)	421 (87.2)	
Yes	128 (13.3)	66 (13.7)	62 (12.8)	
Tumor median (IQR), cm	3.50 (2.50–5.00)	3.60 (2.50–5.00)	3.50 (2.50–4.80)	**<0.01**
TC median (IQR), mmol/L	4.93 (4.31–5.61)	4.80 (4.24–5.47)	5.05 (4.45–5.69)	**<0.01**
TG median (IQR), mmol/L	1.13 (0.89–1.47)	1.09 (0.86–1.42)	1.17 (0.94–1.53)	**<0.01**
PNI median (IQR)	50.67 (47.50–53.84)	49.96 (46.80–53.29)	51.35 (48.60–54.23)	**<0.01**
PLR median (IQR)	113.87 (86.73–152.98)	117.58 (90.13–155.81)	110.47 (84.20–148.47)	**0.05**
SII median (IQR)	418.18 (283.37–634.25)	442.86 (304.06–698.12)	381.86 (249.97–584.23)	**<0.01**

The bold *P* value less than 0.05 was considered statistically significant. TNM, tumor node; IQR, interquartile range; TC, total cholesterol; SII, systemic immune-inflammation index; PNI, prognostic nutritional index; PLR, platelet-lymphocyte ratio; TG, triglycerides.

**TABLE 4:** Univariate and multivariate analysis of overall survival in the matching cohort.

Variables	Univariate analysis		Multivariate analysis	
	HR (95% CI)	P	HR (95% CI)	P
Age (≦60 vs. > 60)	0.77 (0.62–0.95)	**0.01**	0.76 (0.61–0.94)	**0.01**
Gender (male vs. female)	1.49 (1.21–1.82)	**<0.01**	1.37 (1.12–1.69)	**< 0.01**
TNM stage (0/1/2 vs. 3/4)	0.27 (0.22–0.34)	**<0.01**	0.33 (0.26–0.42)	**< 0.01**
Surgical margin (R0 vs. R1/R2)	0.69 (0.46–1.02)	0.07		
Location (upper vs. middle, lower)	1.12 (0.91–1.37)	0.28		
Vascular invasion (no vs. yes)	0.43 (0.34–0.55)	**<0.01**	0.68 (0.52–0.89)	**< 0.01**
Neural invasion (no vs. yes)	0.54 (0.41–0.70)	**<0.01**	0.76 (0.57–1.01)	0.06
Tumor grade (moderate, poorly vs. well)	0.99 (0.81–1.21)	0.92		
Postoperative adjuvant treatment (no vs. yes)	0.86 (0.71–1.06)	0.15		
Tumor diameter (continuous)	1.15 (1.10–1.19)	**<0.01**	1.09 (1.04–1.14)	**< 0.01**
TC (continuous)	1.01 (0.91–1.11)	0.91		
TG (continuous)	0.94 (0.83–1.07)	0.94		
PNI (continuous)	0.97 (0.95–0.99)	**<0.01**	0.99 (0.97–1.01)	0.41
PLR (continuous)	1.00 (1.00–1.00)	**<0.01**	1.00 (1.00–1.00)	0.98
SII (continuous)	1.00 (1.00–1.00)	**<0.01**	1.00 (1.00–1.00)	0.63

The bold *P* value less than 0.05 was considered statistically significant. HR, hazard ratio; CI, confidence interval; TNM, tumor node; SII, systemic immune-inflammation index; PNI, prognostic nutritional index; PLR, platelet-lymphocyte ratio; TC, total cholesterol; TG, triglycerides.

### Sex-stratified survival analysis

We further determined the difference between clinicopathological variables and these prognostic factors between gender by sex-stratified survival analysis. In the univariate Cox analysis, TNM stage, vascular invasion, neural invasion, tumor grade, tumor diameter, and TC were significantly associated with OS in both sexes (*p* < 0.05 for all) (Supplementary Table S1). Furthermore, PNI, PLR, and SII were significantly associated with prognosis in males but not in females. In the multivariable Cox analysis, TNM stage, surgical margin, vascular invasion, neural invasion, tumor grade, tumor diameter, and PNI were independent factors for OS in males (*p* < 0.05 for all). After PSM, TNM stage, vascular invasion, and tumor diameter were independent factors for OS in males (*p* < 0.05 for all) (Table 5). Whether or not PSM was employed, the result showed TNM stage, neural invasion, and TC were independent prognostic factors in females (*p* < 0.05 for all).

**TABLE 5:** Univariate and multivariate analysis of overall survival in ESCC patients stratified by gender after PSM.

Variables	Male (*n* = 483)				Female (*n* = 483)			
	Univariate analysis		Multivariate analysis		Univariate analysis		Multivariate analysis	
	HR (95% CI)	P	HR (95% CI)	P	HR (95% CI)	P	HR (95% CI)	P
Age (≤60 vs. > 60)	0.78 (0.59–1.03)	0.08			0.75 (0.55–1.04)	0.09		
TNM stage (0/1/2 vs. 3/4)	0.27 (0.20–0.37)	**<0.01**	0.35 (0.25–0.48)	**<0.01**	0.27 (0.19–0.38)	<0.01	0.32 (0.22–0.45)	**<0.01**
Surgical margin (R0 vs. R1/R2)	0.76 (0.47–1.21)	0.24			0.72 (0.34–1.54)	0.40		
Location (upper vs. middle, lower)	1.19 (0.91–1.55)	0.20			1.06 (0.78–1.44)	0.71		
Vascular invasion (no vs. yes)	0.45 (0.32–0.63)	**<0.01**	0.60 (0.41–0.88)	**0.01**	0.40 (0.28–0.58)	<0.01	0.71 (0.48–1.05)	0.09
Neural invasion (no vs. yes)	0.65 (0.45–0.93)	**0.02**	0.95 (0.63–1.41)	0.78	0.43 (0.29–0.65)	<0.01	0.58 (0.38–0.87)	**0.01**
Tumor grade (moderate, poorly vs. well)	0.95 (0.73–1.24)	0.70			1.01 (0.74–1.36)	0.97		
Postoperative adjuvant treatment (no vs. yes)	0.94 (0.72–1.23)	0.65			0.76 (0.56–1.03)	0.07		
Tumor diameter (continuous)	1.14 (1.08–1.19)	**<0.01**	1.11 (1.04–1.17)	**<0.01**	1.13 (1.05–1.22)	<0.01	1.06 (0.97–1.15)	0.20
TC (continuous)	0.91 (0.80–1.03)	0.12			1.22 (1.05–1.42)	0.01	1.25 (1.06–1.47)	**0.01**
TG (continuous)	0.95 (0.81–1.11)	0.49			0.97 (0.80–1.17)	0.73		
PNI (continuous)	0.97 (0.95–1.00)	0.06			1.00 (0.97–1.03)	0.98		
PLR (continuous)	1.00 (1.00–1.00)	0.07			1.00 (1.00–1.00)	0.11		
SII (continuous)	1.00 (1.00–1.00)	0.54		0.35	1.00 (1.00–1.00)	0.56		

The bold *P* value less than 0.05 was considered statistically significant. HR, hazard ratio; CI, confidence interval; TNM, tumor node; SII, systemic immune-inflammation index; PNI, prognostic nutritional index; PLR, platelet-lymphocyte ratio; TC, total cholesterol; TG, triglycerides.

### Serum total cholesterol and clinicopathological features in the female cohort

To further elucidate the relationship between TC and the prognosis of ESCC in the female cohort, 487 female patients were separated into two groups according to the optimal cut-offs of TC. Table 6 showed the clinicopathological features of female patients in accordance with TC level. Of all female patients, 352 (72.3%) patients were in the TC-low group and 135 (27.7%) were in the TC-high group. There were significant differences in TG and PNI (*p < 0.05*), but other stated clinicopathological variables were no significant differences between the TC-low group and TC-high group in this study.

**TABLE 6:** Serum total cholesterol and characteristics of the female patients with ESCC.

Characteristics	Low TC(*n* = 352)	High TC (*n* = 135)	*p* value
Age median (IQR)	62.00 (57.25–68.00)	64.00 (58.00–69.00)	0.12
TNM stage			0.51
0	16 (4.5)	4 (3.0)	
I	50 (14.2)	17 (12.6)	
II	124 (35.2)	41 (30.4)	
III	139 (39.5)	60 (44.4)	
IV	23 (6.5)	13 (9.6)	
Tumor grade			0.42
Poorly	152 (43.2)	50 (37)	
Moderate	121 (34.4)	49 (36.3)	
Well	79 (22.4)	36 (26.7)	
Tumor location			0.70
Lower	39 (11.1)	13 (9.6)	
Middle	181 (51.4)	66 (48.9)	
Upper	132 (37.5)	56 (41.5)	
Postoperative adjuvant treatment			0.57
No	204 (58)	82 (60.7)	
Yes	148 (42)	53 (39.3)	
Surgical margin			0.65
R0	342 (97.2)	129 (95.6)	
R1	6 (1.7)	4 (3.0)	
R2	4 (1.1)	2 (1.4)	
Neural invasion			0.81
No	313 (88.9)	119 (88.1)	
Yes	39 (11.1)	16 (11.9)	
Vascular invasion			0.71
No	307 (87.2)	116 (85.9)	
Yes	45 (12.8)	19 (14.1)	
Tumor median (IQR), cm	3.50 (2.50–5.00)	3.30 (2.20–4.50)	0.39

The bold *P* value less than 0.05 was considered statistically significant; TNM, tumor node; IQR, interquartile range; TC, total cholesterol; SII, systemic immune-inflammation index; PNI, prognostic nutritional index; PLR, platelet-lymphocyte ratio; TG, triglycerides.

In the Kaplan–Meier analysis, the high TC level (>5.62 mmol/L) showed a significantly shorter OS (log-rank *p* < 0.01) than the low TC level (≦5.62 mmol/L) (Figure 2C). In addition, Cox regression analysis revealed high TC level was an independent risk factor of ESCC in the female after adjusting for TNM stage, vascular invasion, neural invasion, tumor grade, and tumor diameter. (HR:1.65, 95% CI:1.20–2.28, *p* < 0.01). Furthermore, the predictive performance of TC was calculated by the time ROC curve (Figure 2D). The AUC values of 3-and 5-year OS rates were 0.63 and 0.70, respectively.

### Subgroup analysis by postoperative adjuvant treatment and pathological stage in the female cohort

**FIGURE 3:**
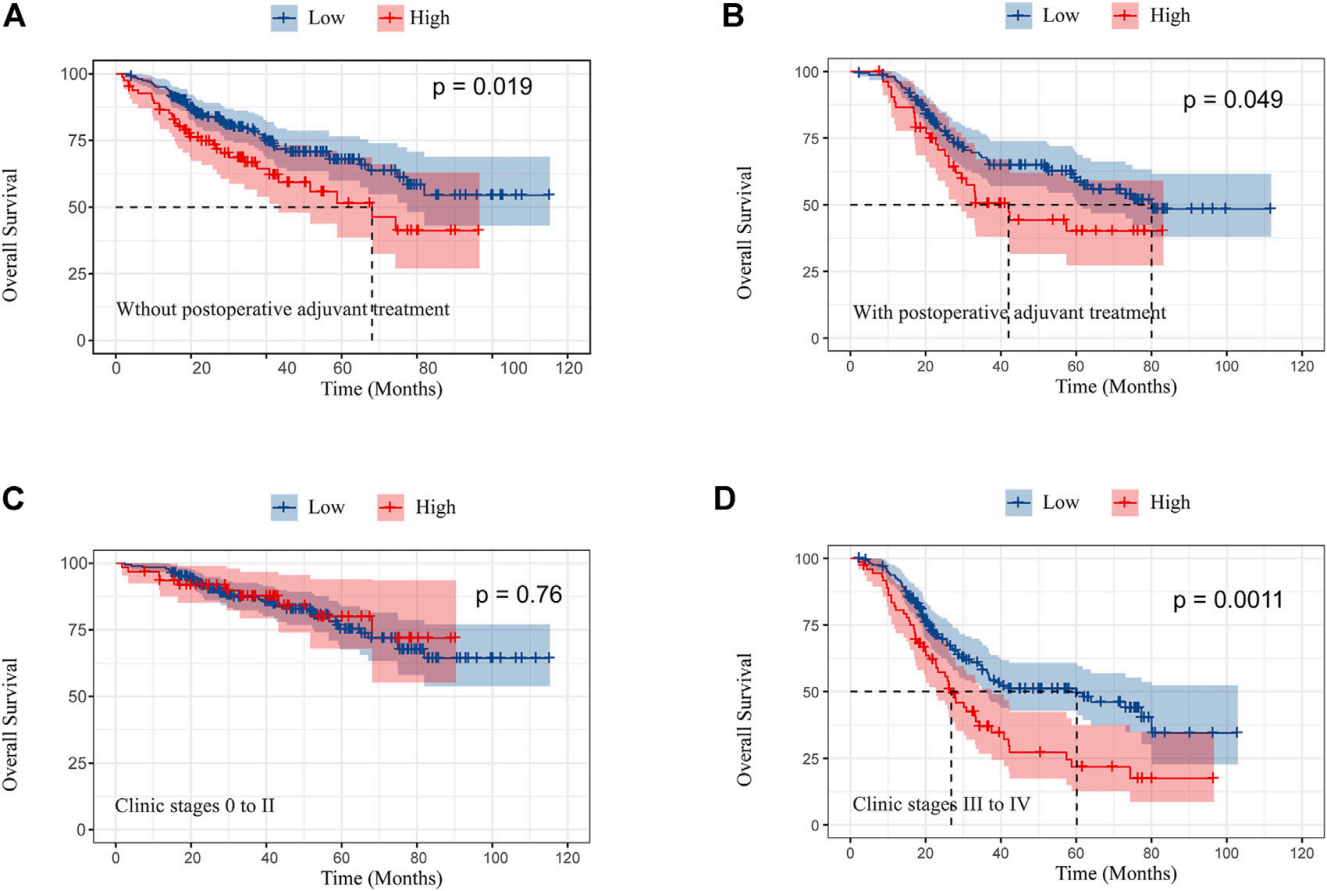
Kaplan–Meier curves of overall survival in female patients without postoperative adjuvant treatment **(A)** and with postoperative adjuvant treatment **(B)**; overall survival in clinic stages 0 to II **(C)** and III and IV **(D)** according to preoperative TC.

In this study, for female patients with or without postoperative adjuvant treatment, Kaplan–Meier analysis showed that OS was significantly better in female patients with low TC levels (*p* < 0.05 for all) (Figures 3A,B). Although OS in stages 0, I, and II was not significant between high and low TC levels, OS in stages III and IV significantly ameliorated in patients with low TC levels (Figures 3C,D).

### Survival analyses of interactions between total cholesterol and other variables in females

**FIGURE 4:**
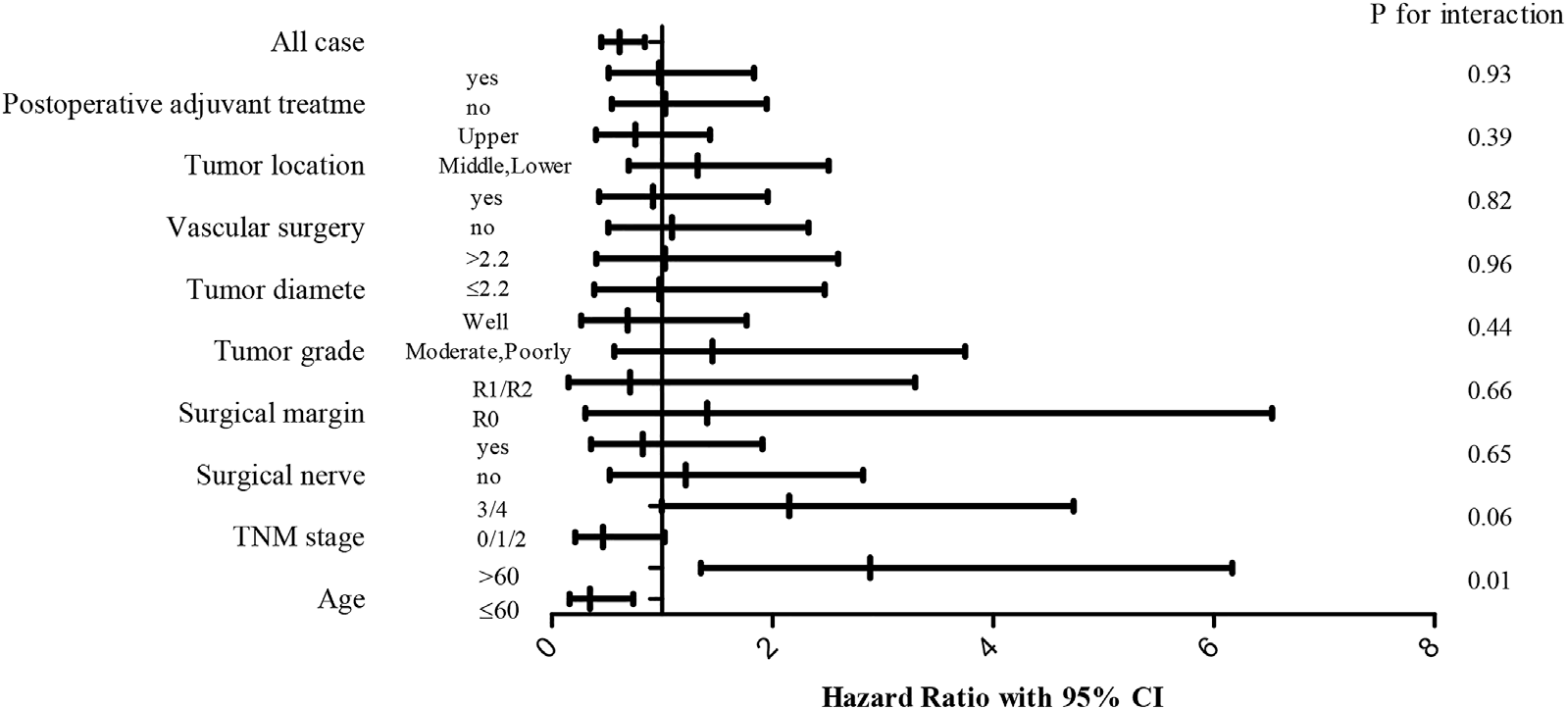
Forest plots of hazard ratio (HR) with 95% CI for each of the stated variables for the female cohort.

We assessed the relationship between TC and other variables to further determine the influence of TC on OS whether affected by any of the clinicopathological variables. The effect of TC was not significantly modified by postoperative adjuvant treatment, tumor location, vascular invasion, tumor diameter, tumor grade, surgical margin, neural invasion, and TNM stage (*p* > 0.05 for all interactions) (Figure 4). Interestingly, it was statistically significant the effect of age on the relationship between TC and prognosis.

### Nomogram model

**FIGURE 5:**
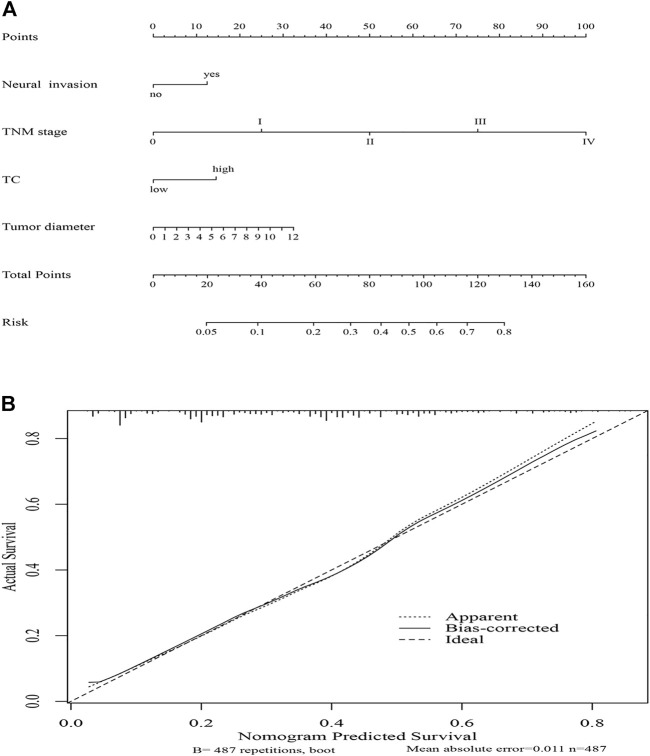
Nomogram model for death risk prediction **(A)**; calibration curve of the nomogram model in the female cohort **(B)**.

To predict the risk for female patients with ESCC, a novel nomogram model was constructed by significant factors, including TNM stage, neural invasion, tumor diameter, and TC (Figure 5A). The C-index was 0.718 (95% CI: 0.679–0.757), indicating good discrimination. Furthermore, the calibration curve of the nomogram for the probability of TC revealed good predictive accuracy between prediction and observation in the female cohort (Figure 5B).

## Discussion

In this study, we focused on the clinical value of PLR, SII, PNI, and serum lipids in 2660 ESCC patients after esophagectomy. In combined survival analysis, the results showed that PNI, PLR, SII, and TC were associated with outcomes in ESCC. Based on a much larger cohort, our results demonstrated that PNI was a powerful prognostic predictor (Xue et al., 2019; Hao et al., 2020). Several pieces of research demonstrated that SII was more promising than the PLR as an independent risk factor in various types of cancer (Hong et al., 2015). Furthermore, Feng et al. (2017) showed the same result in ESCC (Fu et al., 2018; Guo et al., 2019). As a result, neither the SII nor PLR was a powerful prognostic predictor in all patients with ESCC in this study. A report had shown that the level of TC was lower and suggested that a low TC level might be a predictive factor for poor prognosis in esophageal cancer patients who underwent esophageal resection (Chen et al., 2017). In all cohorts, our study showed the same result. However, in sex-stratified survival analysis, the results showed that PNI and SII were related to prognosis in males but not in females. Contrarily, TC was a significant prognostic factor for females not for males. Consequently, when we evaluated the relationship between prognostic predictors and survival outcomes of cancers, we should not only consider the pathological parameters and treatment methods but also consider sex-stratified analysis.

The reproductive systems, hormonal environment, and gene expression were affected by the sex difference. The concentrations of blood biomarkers often vary with sex, age, metabolism, diet, race, and disease status (Blanck et al., 2003). A study showed even though in the same disease, significant differences in the progress of the disease and the response to treatment between sex (Weiss et al., 2006). The applicability of traditional cut-off values previously set for the general population with ESCC might be questionable for determining the physical state in females. Therefore, we suggest the cut-offs and clinic values of prognostic predictors (e.g., PLR, SII, PNI, TC, and other new predictors) should be determined by sex-stratified analysis in future prospective studies.

Sex differences had been consistently observed as a risk factor for esophageal cancer (Cook et al., 2005). For cohorts with or without matching, our study showed the same results sex was confirmed as an independent prognostic factor, and females were associated with better outcomes in ESCC. We assessed the predictive functions of TC in female patients. The AUC values for 3- and 5-year OS rates presented good predictive stability of TC in the female cohort. In subgroup analysis, we found TC was correlated to overall survival when female patients had no adjuvant treatments or received adjuvant treatments. The results confirmed the prognostic predictive value of TC in female patients with or without adjuvant treatments after esophagectomy. Kimura and Sumiyoshi (2007) found a high-cholesterol diet might promote cancer growth and metastasis in cancer-bearing mice. In addition, several studies had found high TC level was a risk factor for cancer metastasis (Sako et al., 2004; Liu et al., 2012; Li et al., 2020). Similarly, a significant survival difference between the low and high TC groups was observed in female patients with stages III and IV. Therefore, controlling TC concentrations might play an important role in metastasis prevention of female patients with ESCC. Consequently, we believe patients may benefit from targeted treatments after adjusting their TC concentrations based on sex-stratified, in addition to routine cancer therapies.

Based on TC, neural invasion, tumor diameter, and TNM stage, we established a predictive nomogram model to predict the probability of death risk for female patients with ESCC. The C-index (0.718) performed well in predicting OS. Meanwhile, good predictive accuracy between prediction and observation was performed by the calibration curve of the nomogram in the female cohort. However, more prospective studies with large sample sizes and more data remain to be confirmed the prognostic role of TC in female patients with esophagectomy in the future.

To our knowledge, this is the first report to comprehensively assess the clinical implication of TC in female patients with ESCC based on retrospective and bioinformatics studies. But there were some limitations. First, because of the single-center retrospective study, a selection bias was inevitable in our study. More multicenter, prospective studies need to be confirmed our results. Second, it was a retrospective study, so TC concentration regarding this analysis was determined at a single time point. Therefore, further investigation on the relationship between TC level and more factors may contribute to establishing the clinical implication of TC as a prognostic predictor in female ESCC patients in the long term.

## Conclusion

In conclusion, a significant survival difference in the female with ESCC between low and high TC groups was confirmed in this study. High TC level might be an independent prognostic factor in the female with ESCC. Sex as a strong human variable, we suggest the cut-offs and clinic values of prognostic predictors (e.g., PLR, SII, PNI, TC, and other new predictors) should be determined by sex-stratified analysis in future prospective studies. In addition, it provided some new ideas for choosing cancer treatment methods by sex-stratified analysis further.

## Data Availability

The raw data supporting the conclusion of this article will be made available by the authors, without undue reservation.

## Abbreviations

CI, confidence interval; ESCC, Esophageal squamous cell cancer; HR, hazard ratio; IQR, interquartile range; OS, overall survival; PLR, platelet to lymphocyte ratio; PNI, prognostic nutritional index; PSM, propensity score matching; ROC, receiver operating characteristic; SD, standard deviations; SII, systemic immune-inflammation index; TC, total cholesterol; TNM, tumor node.
