# Supplementary material for: Prognostic value of nutritional and inflammatory indicators in females with esophageal squamous cell cancer: A propensity score matching study
Source: Front Genet. 2022 Oct 31;13:1026685. doi: 10.3389/fgene.2022.1026685 (PMC9659636; doi:10.3389/fgene.2022.1026685)
Supplement: Supplementary file 1 [file Table1.docx]

**Supplements**

There were a total of 2,660 patients with ESCC. 2173 (82%) were male and 487 (18%) were female. Before PSM, the differences of clinicopathologic variables and prognostic factors between gender were presented in the Table S1.

**Table S1 Univariate and multivariate analysis of overall survival in ESCC patients stratified by gender before PSM**

| **Variables** | **Male (n=2173)** | | | | **Female (n=487)** | | | |
| --- | --- | --- | --- | --- | --- | --- | --- | --- |
|  | **Univariate Analysis** | | **Multivariate Analysis** | | **Univariate Analysis** | | **Multivariate Analysis** | |
|  | HR (95% CI) | P | HR (95% CI) | P | HR (95% CI) | P | HR (95% CI) | P |
| Age (<=60 vs >60） | 0.96(0.85-1.08) | 0.49 |  |  | 0.75(0.54-1.04) | 0.08 |  |  |
| TNM stage (0/1/2 vs 3/4) | 0.33(0.28-0.37) | **<0.01** | 0.38(0.33-0.45) | **<0.01** | 0.26(0.19-0.37) | **<0.01** | 0.32(0.22-0.45) | **<0.01** |
| Surgical margin (R0 vs R1/R2) | 0.57(0.45-0.71) | **<0.01** | 0.72(0.57-0.90) | **<0.01** | 0.72(0.33-1.53) | 0.39 |  |  |
| Location (upper vs middle, lower) | 1.17(1.02-1.34) | **0.03** | 1.10(0.96-1.27) | 0.19 | 1.05(0.77-1.42) | 0.77 |  |  |
| Vascular invasion (no vs yes) | 0.58(0.50-0.67) | **<0.01** | 0.73(0.63-0.85) | **<0.01** | 0.42(0.29-0.61) | **<0.01** | 0.73(0.50-1.09) | 0.12 |
| Neural invasion (no vs yes) | 0.68(0.59-0.79) | **<0.01** | 0.86(0.74-0.99) | **0.04** | 0.43(0.29-0.64) | **<0.01** | 0.59(0.39-0.89) | **0.01** |
| Tumor grade (moderate, poorly vs well) | 1.42(1.20-1.67) | **<0.01** | 1.24(1.04-1.47) | **0.01** | 1.66(1.05-2.63) | **0.03** | 1.49(0.94-2.37) | 0.10 |
| Postoperative adjuvant treatment  (no vs yes) | 1.10(0.98-1.24) | 0.11 |  |  | 0.78(0.57-1.05) | 0.10 |  |  |
| Tumor diameter (continuous) | 1.11(1.09-1.14) | **<0.01** | 1.08((1.05-1.11) | **<0.01** | 1.13(1.05-1.22) | **<0.01** | 1.06(0.98-1.16) | 0.16 |
| TC (continuous) | 0.91(0.86-0.97) | **0.01** | 0.95(0.89-1.02) | 0.14 | 1.23(1.06-1.42) | **0.01** | 1.28(1.09-1.50) | **<0.01** |
| TG (continuous) | 0.94(0.87-1.02) | 0.14 |  |  | 0.96 (0.80-1.17) | 0.71 |  |  |
| PNI (continuous) | 0.97(0.96-0.99) | **<0.01** | 0.99(0.97-1.00) | **0.04** | 1.00(0.97-1.03) | 0.99 |  |  |
| PLR (continuous) | 1.00(1.00-1.00) | **0.01** | 1.00(1.00-1.00) | 0.47 | 1.00(1.00-1.00) | 0.10 |  |  |
| SII (continuous) | 1.00(1.00-1.00) | <0.01 | 1.00(1.00-1.00) | 0.51 | 1.00(1.00-1.00) | 0.53 |  |  |

The bold *P* value less than 0.05 was considered statistically significant. HR, hazard ratio; CI, confidence interval; TNM, tumor node; SII, systemic immune-inflammation index; PNI, prognostic nutritional index; PLR, platelet-lymphocyte ratio; TC, total cholesterol; TG, triglycerides
